# Thermal Ablation of Hypervascular Liver Tumors After Selective Intra-Arterial Lipiodol Injection (SIALI): A Technical Narrative Review of Technique, Institutional Variations, and Published Evidence

**DOI:** 10.3390/cancers18142209

**Published:** 2026-07-09

**Authors:** Kiyon Naser-Tavakolian, Abin Sajan, Alyssa Knight, Nikhil Jacob, Stephen P. Reis, Ahmed Abdelal, Binta Patel, Michael A. Clifton, Muhammad Usman Shahid, Kirema Garcia-Reyes, Leigh Casadaban, Maarten L. J. Smits, Zachary T. Berman, Venkatesh P. Krishnasamy

**Affiliations:** 1 Vascular and Interventional Radiology, Columbia University, New York, NY 10027, USA; 2 Vascular and Interventional Radiology, University of Alabama Birmingham, Birmingham, AL 35294, USA; 3 College of Osteopathic Medicine, Kansas City University, Kansas City, MO 64106, USA; 4 Guerbet, Princeton, NJ 08540, USA; 5 Vascular and Interventional Radiology, University of Minnesota Medical Center, Minneapolis, MN 55455, USA; 6 Department of Interventional Radiology, University of Miami Miller School of Medicine, Miami, FL 33136, USA; 7 Vascular and Interventional Radiology, Icahn School of Medicine at Mount Sinai, New York, NY 10029, USA; 8 Vascular and Interventional Radiology, University of Colorado Anschutz Medical Campus, Aurora, CO 80045, USA; 9 Department of Radiology and Nuclear Medicine, University Medical Center Utrecht, 3584 CX Utrecht, The Netherlands; 10 Vascular and Interventional Radiology, University of California San Diego, San Diego, CA 92037, USA

**Keywords:** hepatocellular carcinoma, thermal ablation, lipiodol, radiofrequency ablation, microwave ablation, interventional radiology, image-guided ablation, transarterial embolization, liver tumor, ablation margin

## Abstract

Purpose: Percutaneous thermal ablation of hypervascular liver tumors is limited by lesion conspicuity on conventional imaging, challenges with ablative margin assessment, and unequal global access to advanced technologies such as ablation confirmation software and radioembolization. This review describes the selective intra-arterial lipiodol injection (SIALI) technique, its technical variations across institutions, and summarizes published clinical outcomes for SIALI-guided thermal ablation of hepatic malignancies. Methods: A narrative review was conducted through structured searches of the PubMed, Embase, and Cochrane databases using terms including “selective intra-arterial lipiodol injection,” “lipiodol-guided ablation,” “SIALI,” and “hepatic arterial lipiodol.” Studies were selected based on relevance to SIALI technique, clinical outcomes, and comparator techniques, including hepatic arteriography with C-arm CT-guided ablation (hepACAGA) and ablation confirmation software. Full-text peer-reviewed articles were included; conference abstracts and letters were excluded. No formal PRISMA-compliant inclusion or exclusion criteria were applied, consistent with the narrative review format. Results: Seven studies were identified, encompassing six retrospective cohort studies and one prospective randomized controlled trial, collectively comprising over 900 patients treated across institutions in Asia, Europe, and the United States. Across heterogeneous patient populations and technical approaches, SIALI-guided thermal ablation was associated with improved local tumor recurrence rates, local recurrence-free survival, and overall survival compared with conventional ultrasound- or CT-guided ablation alone in selected studies where comparator data were available, with favorable technical success rates in lesions otherwise occult on standard imaging. Conclusion: SIALI appears to be a technically feasible and potentially useful adjunct technique that may improve lesion conspicuity and enable real-time ablative margin assessment in the treatment of hypervascular liver tumors, particularly in settings without access to ablation confirmation software. The available evidence is predominantly retrospective, and prospective validation is needed before broad conclusions regarding oncologic outcomes can be drawn.

## Simple Summary

Percutaneous thermal ablation is an established treatment for liver tumors, but its effectiveness can be limited when tumors are difficult to visualize on standard imaging. Selective intra-arterial Lipiodol injection (SIALI) is a technique in which iodized oil is delivered directly into the hepatic artery supplying a tumor, causing preferential retention within hypervascular tumors and rendering them visible on fluoroscopy and computed tomography. This technical narrative review describes how SIALI is performed, its technical variations across institutions, and summarizes the available published clinical evidence. Existing studies, predominantly retrospective and focused on hepatocellular carcinoma, suggest that SIALI-guided ablation may improve local tumor control compared to conventional imaging guidance alone. The technique may be particularly valuable in centers without access to advanced ablation confirmation software or navigational platforms. Prospective studies are needed to confirm these findings and to establish standardized protocols.

## 1. Introduction

Primary liver cancer is the sixth most common cancer worldwide and one of the leading causes of overall cancer-related death globally [1]. Despite well-known risk factors and advancements in treatment strategies, it is estimated that the incidence of liver cancer will increase by about 40% by 2040 [2]. Hepatocellular carcinoma (HCC) accounts for around 80% of primary liver cancers, and the etiology of the disease has shifted from virus-related (Hepatitis B or C) to underlying metabolic disease (metabolic-associated steatotic liver disease or MASLD) in developed countries [3]. Metastatic liver cancer, which is more common than primary liver cancer, is prevalent in up to 5–15% of all cancer patients, with the most common primary sites including breast, lung, and colorectal cancer [4].

The treatment approach for HCC depends on various factors, including tumor size, location, liver function, comorbidities, and the patient’s functional status. The Barcelona Clinic Liver Cancer staging system (BCLC) classification is utilized to categorize HCC in five categories with specific treatment recommendations [5]. While surgical resection and liver transplantation were traditionally considered the curative treatment options, curative intent with locoregional therapies (thermal ablation, ablative Y90) is also supported in treatment algorithms. Treatments are selected based on lesion and patient characteristics [6,7,8,9].

Percutaneous ablation is a minimally invasive therapeutic option that, with adequate margins, offers effective local tumor control for liver tumors less than 3 cm. Thermal ablation techniques, including radiofrequency ablation (RFA), microwave ablation (MWA), and cryoablation (CRA), rely on the application of extreme heat or cold to induce tumor necrosis [10]. To achieve appropriate targeting and provide complete ablation of the tumor and its margin, imaging guidance techniques such as computed tomography (CT) with or without ultrasound (US), CT fluoroscopy, and fused intraprocedural CT imaging are frequently used. These techniques have limitations, however, in visualizing certain liver tumors, particularly those in difficult anatomical locations, those with poor intrinsic contrast, and also in patients with increased BMI [11,12,13]. Additionally, there is positive and promising early data for ablation confirmation software utilizing software-assisted ablation margin evaluation, but access to these technologies is not ubiquitous due to cost and regulatory approvals [14,15,16,17].

Combination approaches with angiography and CT for thermal ablation have been previously studied. Variations using hepatic angiography and cone beam CT (hepACAGA technique) have been more recently utilized for flexibility and improved lesion conspicuity, as well as treatment outcomes compared to standard CT-guided ablation alone [18]. This technique is especially relevant given the hurdles of transferring a patient from the angiography suite to the CT scanner in real time. While tumor targeting can still be performed with US using the hepACAGA technique, needle guidance software in the angiographic suite can also be utilized. In addition, the marked upside of utilizing a hepatic arterial catheter, whether with cone-beam CT (CBCT) or CT, is the ability to perform repeated, low-contrast-dose, enhanced CBCTs (or CTs) for tumor targeting and ablation confirmation.

Selective intra-arterial lipiodol injection (SIALI technique) into the tumor, performed prior to thermal ablation, is a technique that utilizes the radiopacity of lipiodol, an agent that contains iodine organically combined with ethyl esters of fatty acids of poppy seed oil, to mark the tumor, guide thermal ablation probe placement, and ensure adequate ablative margins [19]. This allows immediate ablation using the hepACAGA technique, as described above, or with CT guidance, whether it be same day or on a subsequent day. A distinct advantage of the SIALI technique is its ability to render otherwise occult liver lesions targetable for percutaneous ablation, particularly tumors not visible on ultrasound or non-contrast CT, addressing a potential limitation in image-guided therapies [20,21]. As a result, SIALI can add further flexibility and options within this treatment paradigm. This review aims to describe the SIALI technique and its variations across institutions, summarize outcomes from published studies utilizing SIALI across multiple international groups, and contextualize SIALI as a complementary and potentially more accessible tool within the locoregional therapy paradigm, particularly for centers without access to ablation-confirmation software or radioembolization.

## 2. Literature Search

This manuscript is structured as a narrative review. A search of PubMed, Embase, and Cochrane databases was performed using the terms “selective intra-arterial lipiodol injection”, “lipiodol-guided ablation”, “SIALI”, “lipiodol thermal ablation”, and “hepatic arterial lipiodol”. No formal PRISMA-compliant inclusion or exclusion criteria were applied, consistent with the narrative review format. Full-text peer-reviewed articles were included; conference abstracts and letters were excluded. The absence of formal bias assessment tools represents a limitation of the narrative review methodology and is acknowledged as such. Studies were selected based on relevance to the SIALI technique, clinical outcomes of lipiodol-guided thermal ablation, and comparator techniques, including hepACAGA and ablation confirmation software. Given the limited and heterogeneous nature of the published evidence, a systematic review or meta-analysis was not feasible.

## 3. Technique

There is variability in the described technique depending on the available technology, operator preference, and hospital workflow. In general, arterial access is obtained via the common femoral or radial artery. The common hepatic artery is then selected under fluoroscopic guidance and digital subtraction angiography (DSA); contrast-enhanced CBCT is then performed to identify the target arteries that supply the tumor. A coaxial microcatheter system is then introduced into the targeted arterial branches selectively, and after confirmation, Lipiodol can be injected alone or emulsified with other agents, depending on the operator’s preference. Emulsification agents include saline, contrast, or chemotherapeutic agents. Unlike water-soluble iodinated contrast agents, Lipiodol allows for prolonged tumor visualization and is more densely retained within hypervascular liver tumors due to its oily nature and preferential uptake within tumor neovasculature. In HCC specifically, this retention is known to persist, enabling visualization of the target lesion for 4 weeks or longer, whereas conventional contrast agents rapidly wash out, limiting their utility for procedural guidance and delayed imaging assessment [22,23]. This preferential retention reflects the underlying tumor biology: hypervascular HCC nodules receive arterial supply without a functional portal venous contribution and lack normal Kupffer cell activity within the tumor itself, resulting in delayed clearance of iodized oil. Hypervascular metastases such as neuroendocrine tumors retain Lipiodol by a similar mechanism. Hypovascular tumors, including most colorectal liver metastases, show poor or unreliable Lipiodol uptake, which limits the applicability of SIALI in these histologies. It should be noted, however, that ultra-selective catheterization and injection technique may improve Lipiodol uptake even in relatively hypovascular lesions, as demonstrated by Miyayama et al. in early-stage HCC [24]. Additionally, lipiodol’s high radiopacity enhances the lesion detection of small satellite or daughter nodules that may otherwise be occult, thereby improving treatment planning [22].

Following intra-arterial infusion, thermal ablation is often performed under CBCT or CT guidance. US can also be used for visualization and targeting of the lesion; however, it is essential to recognize that Lipiodol within the tumor exhibits varied appearances on US and can change the appearance from US evaluation of the lesion prior to utilization of the SIALI technique [19]. It is also worth noting the complementary role of contrast-enhanced ultrasound (CEUS) in liver ablation workflows. CEUS allows real-time characterization of tumor vascularity and immediate post-ablation viability assessment without radiation exposure and is used in European and Asian centers for HCC and hypervascular metastases [25]. In centers where CEUS guidance is routinely available, and lesions are detectable on CEUS, its use may reduce the need for Lipiodol-based marking. SIALI and CEUS are not mutually exclusive, and SIALI provides iodine-based tumor marking visible on fluoroscopy and CT/CBCT that persists beyond a single imaging session, which may be useful as a complement to CEUS in hybrid suite workflows or for lesions occult on CEUS. The choice of ablation method (RFA, MWA, or CRA) depends on tumor size, tumor type, location, operator preference, and availability of technology. While intra-arterial delivery of lipiodol and thermal ablation of the lesion can be performed sequentially at separate times with a short interval, single-session therapy is safe and feasible. Workflow also depends on locally available technology and patient flow, as a separate angiography and CT suite may prove challenging for single-session intervention. However, the added value of a multimodality suite with both multidetector CT (MDCT) and angiography is well known [18,22,26].

Multiprobe configurations or overlapping ablations with a single probe may be used for larger tumors, especially those larger than 3 cm and with heat-based devices. Real-time US, CBCT, or CT imaging can be used to monitor the ablation zone in vivo. Of these, US monitoring has been shown to be a more accurate correlation with the radiologic and pathologic ablation zone [27,28]. If performed in a single session, repeated low contrast dose injections from the hepatic arterial catheter can also help monitor and guide treatment. However, predicting ablation zone size based solely on the manufacturer’s recommendation based on time and power is not advisable and has been repeatedly shown to be inaccurate [29,30,31].

A unique advantage of the SIALI technique, regardless of whether it is used with CT or CBCT, is the ability to visualize the ablation zone simultaneously with the stained tumor on post-ablation contrast-enhanced imaging. Even without software registration of the ablation zone margin, visual confirmation with manual measurements can be performed to help ensure an adequate ablation margin. This workflow offers a different and improved solution from traditional visual assessment on post-ablation intravenous contrast-enhanced MDCT, where the underlying tumor is not visualized and margins are estimated based on landmarks and the expected location of the original tumor, or lack of residual tumor contrast enhancement. This is especially important for centers without ablation confirmation software platforms.

## 4. Suggested Procedural Framework

Based on the institutional approaches described in the reviewed literature and the authors’ combined experience, the following step-by-step framework is offered to facilitate reproducibility and future prospective study design. This represents a suggested workflow rather than a validated evidence-based protocol, and institutional variation in equipment, patient flow, and operator expertise will necessarily influence implementation.

(1) Patient selection: Confirm hypervascular tumor histology (HCC or hypervascular metastasis), adequate hepatic and renal function, absence of iodine hypersensitivity, and availability of simultaneous or sequential CT suite access, hybrid angiography-CT suite, or a dedicated CBCT-capable angiography room. (2) Pre-procedural imaging: Review cross-sectional imaging to identify target lesion, assess lesion vascularity, and plan arterial access and catheterization approach. (3) Vascular access and hepatic angiography: Obtain femoral or radial arterial access; perform common hepatic and selective lobar/segmental DSA with CBCT or CT to identify tumor-feeding arteries. (4) Selective catheterization and Lipiodol administration: Advance coaxial microcatheter to the most selective position supplying the tumor; inject Lipiodol alone or emulsified with saline or chemotherapy per institutional protocol. Inject slowly under fluoroscopic monitoring. (5) Post-injection CBCT or CT: Confirm intratumoral Lipiodol retention and identify any satellite nodules. Assess adequacy of staining before proceeding. (6) Ablation probe positioning: Under CBCT, CT, or US guidance, advance the ablation probe(s) targeting the Lipiodol-stained tumor with preplanned overlapping ablation zones where appropriate to achieve a 0.5 cm–1.0 cm circumferential margin. (7) Ablation and real-time monitoring: Perform thermal ablation per device protocol; use repeat low-dose contrast-enhanced CBCT or CT from the indwelling arterial catheter to monitor the evolving ablation zone as needed if possible. (8) Post-ablation margin assessment: Acquire contrast-enhanced CBCT or CT immediately post-ablation; visually confirming that the ablation zone encompasses the Lipiodol-stained tumor with adequate margin. Manual measurements may be performed where software platforms are unavailable. (9) Follow-up imaging: Schedule cross-sectional follow-up imaging (contrast enhanced multiphase CT or MRI) per institutional protocol, typically at 4–6 weeks and then every 3 months post procedure.

SIALI requires proficiency in both hepatic angiography and image-guided ablation and is therefore operator-dependent. Formal learning curve data are not available in the published literature, but the technique is most appropriately performed by interventional radiologists experienced in selective hepatic arteriography and percutaneous liver ablation. This operator dependency is a practical barrier to widespread adoption and warrants acknowledgment when interpreting outcomes from expert referral centers.

**Figure 1:**
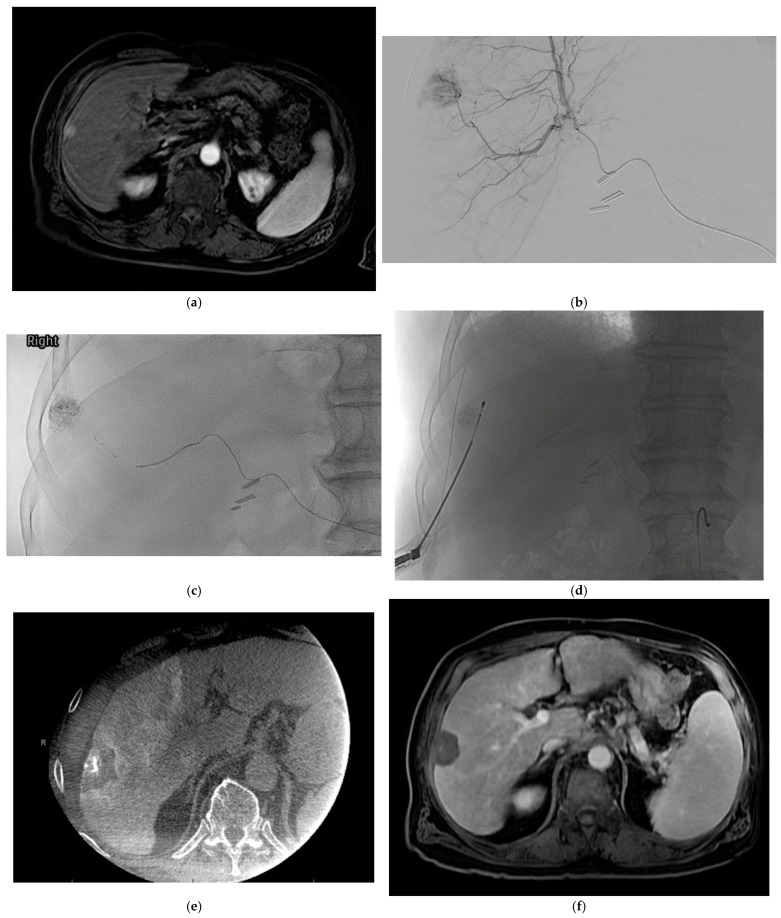
(**a**–**f**) 75 year old male with metabolic dysfunction-associated steatotic liver disease cirrhosis with a 1.5 cm subcapsular hepatocellular carcinoma in segment 8. Not a resection candidate due to comorbidities. (**a**) Arterial phase axial MRI image showing the hypervascular HCC in segment 8. (**b**) Digital subtraction angiography showing the hypervascular lesion. (**c**) Fluoroscopic image acquired within 5 min of lipiodol injection initiation. (**d**) CBCT source image acquired in the same session prior to ablation showing the ablation needle positioned within the medial margin of the lesion and a second needle more laterally used for hydrodissection. (**e**) Contrast-enhanced CBCT acquired immediately post-ablation in the same session with an ablative margin. (**f**) Venous phase MRI 7 months post ablation showing a nonviable lesion.

**Figure 2:**
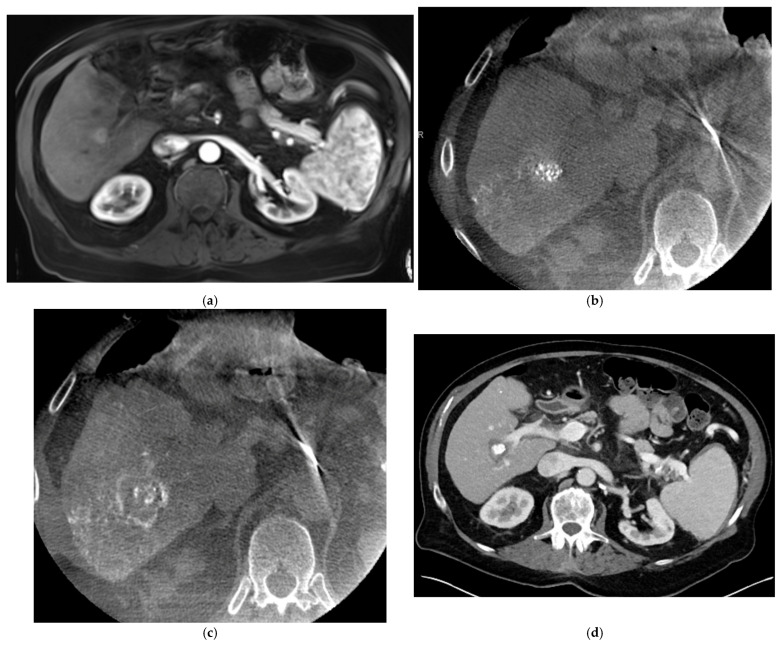
(**a**–**d**) 70 year old male with hepatitis C cirrhosis with 1.6 cm hepatocellular carcinoma in segment 5. The patient was not a surgical or transplant candidate due to comorbidities. (**a**) Pretreatment axial arterial phase MRI showing an enhancing HCC in segment 5. The lesion was invisible on US. (**b**) CBCT acquired in the same session following Lipiodol injection. (**c**) Contrast-enhanced CBCT immediately post-ablation in the same session showing an ablative margin. (**d**) Contrast-enhanced CT 4 months post-ablation with nonviable lesion.

**Figure 3:**
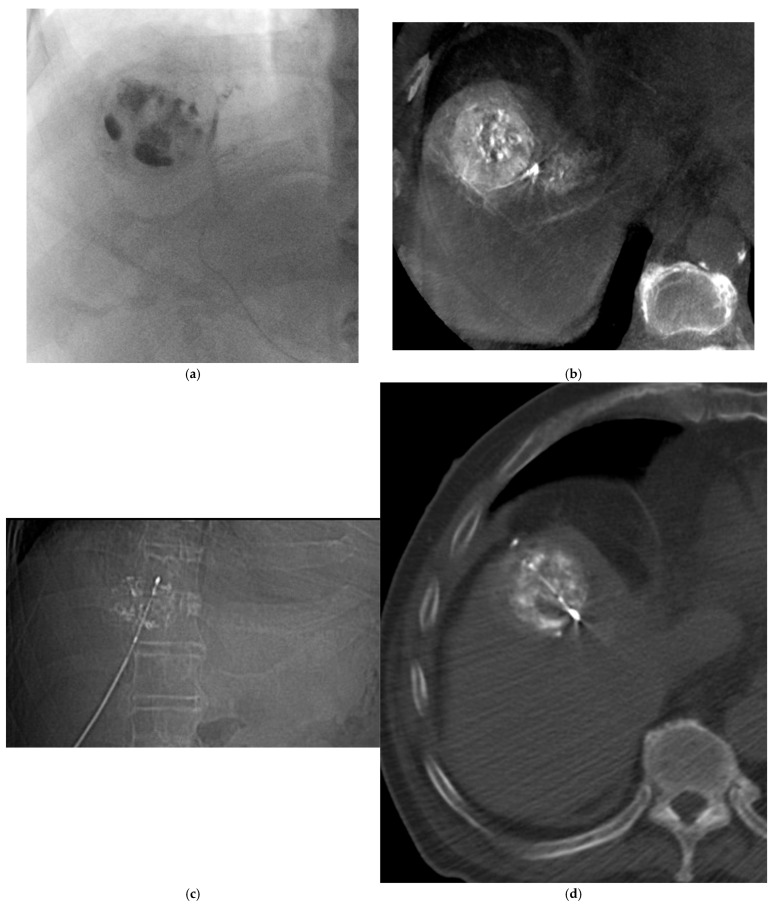

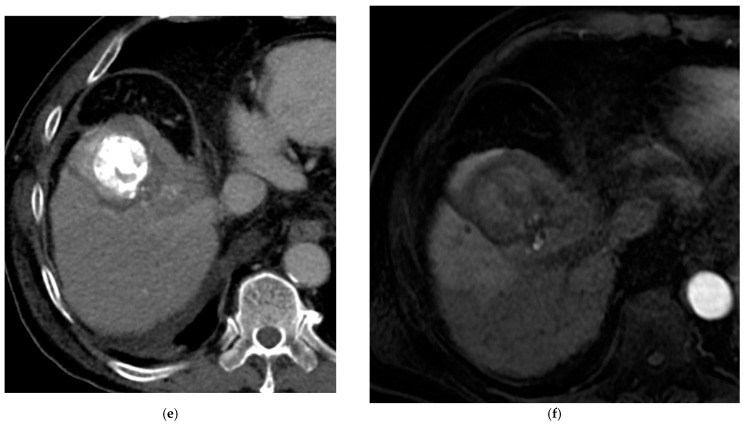
(**a**–**f**) 63-year-old male with a history of hepatitis C cirrhosis with a 4.2 cm hepatocellular carcinoma. Note: Figure 3 illustrates a cTACE/ablation combined workflow in which Lipiodol-based tumor marking occurs as part of conventional TACE, representing one institutional variation in the broader SIALI concept. (**a**) Transarterial chemoembolization (cTACE) of a large 4.2 cm hepatocellular carcinoma (HCC) in segment 8, showing staining of the tumor with Lipiodol. (**b**) CBCT was acquired in the same session following cTACE injection. (**c**) CT localizer the next day after cTACE, showing the ablation probe overlapping the lipiodol-stained lesion. (**d**) Intraprocedural axial CT during ablation. (**e**) Contrast-enhanced CT immediately post-ablation with a visible margin. (**f**) Arterial phase MRI 3 months post-treatment showing nonviable tumor.

Representative cases illustrating the SIALI technique are presented in Figure 1, Figure 2 and Figure 3.

## 5. Current Evidence

For consistency across the studies reviewed, the following definitions apply as used in this narrative: local tumor recurrence (LTR) refers to new enhancement within or immediately adjacent to the ablation zone on follow-up imaging; local recurrence-free survival (LRFS) refers to time from the procedure to LTR or death; local tumor progression (LTP) refers to progressive growth of residual or recurrent disease at the treated site; overall survival (OS) refers to time from procedure to death from any cause; and recurrence-free survival (RFS) refers to time to any recurrence (local or distant) or death. It should be noted that individual studies used these terms variably and with differing measurement criteria; direct cross-study comparisons of outcome rates should therefore be interpreted with caution.

The published literature on SIALI-guided thermal ablation consists of a modest but growing body of evidence. A total of seven studies were included for review, encompassing six retrospective cohort studies and one prospective randomized controlled trial. These studies collectively include over 900 patients treated across institutions in Asia, Europe, and the United States. Patient populations varied in tumor etiology (HCC and metastatic disease), lesion size, and lipiodol preparation (lipiodol alone vs. emulsified formulations), reflecting the inherent heterogeneity of real-world practice. A summary of the studies included is presented in Table 1 and Table 2.

Before summarizing the individual studies, it is important to distinguish the mechanistic basis of the approaches represented in these publications, as they are not homogeneous. Pure SIALI (Lipiodol injection alone for tumor marking) primarily improves lesion conspicuity and enables real-time ablation probe targeting and visual margin assessment. Bland transarterial embolization (TAE) combined with ablation adds an embolic effect that may reduce tumor vascularity, attenuate heat-sink, and contribute to tumor necrosis independently of the ablation [33]. Conventional TACE (cTACE) with Lipiodol-drug emulsion further introduces a chemotherapeutic effect in addition to embolization. Hepatic arteriography with C-arm CT-guided ablation (hepACAGA) uses the arterial catheter for imaging guidance and repeated contrast-enhanced CBCT without necessarily relying on Lipiodol for tumor marking [18]. The outcome benefits reported across the studies below may reflect one or more of these mechanisms, and attribution of improved outcomes solely to Lipiodol-guided targeting would be an oversimplification in studies involving TAE or TACE components. This heterogeneity is acknowledged as a key limitation of the current evidence base.

The unique uptake pattern and retention of Lipiodol in hypervascular liver tumors have been traditionally utilized in TACE to monitor treatment delivery and tumor response [38]. These properties make Lipiodol an optimal imaging marker for ablation guidance. Wu et al. compared the effectiveness of SIALI with C- guided RFA versus US-guided RFA in 320 propensity score-matched patients with HCC retrospectively. All patients were within Milan criteria and treated with curative intent. In the SIALI group, lipiodol was injected alone, and ablation was performed the same day. Over 80% of patients in each group had tumors less than 3 cm and had only 1 lesion treated. Technical success rates were 99.4% and 96.3% for the SIALI and US group, respectively (*p* = 0.121). Local tumor recurrence rates at 1, 2, and 3 years in the SIALI group were 4.4%, 6.9%, and 7.5%, which were significantly lower than the US group (14.4%, 16.3%, and 16.3%, *p* = 0.002, 0.009, and 0.016, respectively). Recurrence-free survival (RFS) was also significantly improved, but overall survival (OS) was not. There was also specifically improved RFS with subdiaphragmatic and subcardiac lesions, demonstrating that challenging locations may benefit from lipiodol guidance. There were no significant differences in adverse events between the two groups [36].

Kobe et al. retrospectively reviewed 18 patients with 20 total lesions that underwent SIALI plus the same-session ablation with RFA, MWA, or CRA. All lesions were less than 3 cm and invisible under US and noncontrast CT. Additionally, 25% were primary HCC. Lipiodol emulsified with saline was utilized for selective injection, and a 5 mm ablative margin was targeted. There was 100% technical success rate and no local recurrence rate observed at a mean follow-up of 3 years, again demonstrating the importance of this technique in difficult-to-target lesions. All interventions were performed in a hybrid angiography MDCT suite. One complication (SIR adverse event severity scale 4) was noted, which was a cardiac arrest due to hormone discharge during ablation of a paraganglioma metastasis. In-hospital mortality in this study was 0% [19]. Takaki et al. also evaluated SIALI for US-invisible lesions retrospectively. In 67 patients with 150 HCCs (mean diameter 1.3 cm), CT fluoroscopy-guided RF ablation was performed within one week of injection of lipiodol, along with a 5-mm ablative margin target. Primary and secondary technical success were 97.3% and 100%. Local tumor progression was 5.3% at 5 years. There was no change in Child Pugh score or mortality, and 6 major complications were recorded (hemorrhage, portal thrombosis, and pneumothorax) [37].

Tan et al. retrospectively reviewed 198 international HCC patients at three institutions who underwent combination SIALI with CT-guided thermal ablation, MWA, or RFA. Selective injection was performed with Lipiodol alone or Lipiodol emulsified with epirubicin and doxorubicin. An ablation margin of 5 mm was targeted. The lipiodol retention pattern was evaluated pre-ablation and classified as complete vs. incomplete. The resultant propensity score matched analysis revealed that HCC with complete lipiodol retention was associated with better local recurrence-free survival (LRFS) compared to the incomplete retention group. The median LRFS was 1279 days (95% CI 821–1746 days) in the complete retention group and 819 days (95% CI 521–1117 days) in the incomplete retention group, which was statistically significant (*p* = 0.030). In the subgroup analyses, this was also shown when ablation was performed less than 1 month from SIALI and in lesions less than 3 cm. While complete lipiodol retention was also shown to have statistically significantly improved LRFS in the TACE subgroup, this was not shown in the lipiodol-alone or the perivascular lesions subgroups. Given high rates of response with thermal ablation alone, these findings may point to potential benefits of the SIALI technique. There was, however, no difference in overall survival between the groups. Adverse events were not reported in this study [35].

Huang et al. retrospectively reviewed 118 HCC (270 total tumors) patients with residual or recurrent disease less than 3 cm. Two groups were compared: RFA only and RFA after transarterial embolization (TAE). Lipiodol alone was injected via the hepatic artery, and ablation was performed with MDCT, aiming for a 5 mm ablative margin. Combination therapy was not always performed on the same day. The technical success rate was 100% for the 270 targeted tumors. Objective response rates (ORR) by mRECIST were significantly higher (*p* = 0.008), and local tumor progression rates were significantly lower (*p* = 0.016) in the combination therapy group. The ORR was 96.61% in the combination group, while being 79.66% in the RFA group. The median time to local tumor progression was 9.6 months vs. 4.8 months, respectively. The major complication rate was 5.08% and 3.39% in the TAE + RFA and RFA alone groups, respectively. These were pneumothorax requiring chest tube (2 cases in TAE + RFA, and 1 case in RFA alone) and massive hemorrhage (1 in each group). The authors purported multiple mechanisms to suggest causation for combination therapy improving outcomes, such as better tumor demarcation, reduced heat sink via vascular occlusion by lipiodol, and increased identification of additional lesions within the liver [33].

Adwan et al. retrospectively evaluated 112 HCC patients split into two groups: transarterial embolization (TAE) plus MWA versus MWA alone. TAE was performed selectively with Lipiodol alone. MWA was performed under CT guidance and not in the same session as TAE. Mean tumor diameter was 1.9 cm and 2 cm in the TAE/MWA and MWA groups, respectively. Complete ablation, defined as having a 5 mm ablative margin, was accomplished in 100% of the TAE/MWA group and 97% of the MWA group, which was not statistically significant. Local tumor progression and progression-free survival were also not significantly different, but, interestingly, 12, 24, and 36-month overall survival rates were statistically significant in favor of the combination group again (97.4%, 80%, and 75.5% vs. 83.7%, 63.8%, and 51.9%—*p* = 0.009). No adverse events were noted in either group [32].

Although TACE-combined ablation represents a distinct treatment paradigm, the Peng et al. prospective randomized controlled trial warrants inclusion here, given that lipiodol-based arterial infusion was integral to the technique and lipiodol staining was utilized as a treatment response marker on follow-up imaging. This overlaps mechanistically with the SIALI approach described in this review. Peng et al. evaluated the long-term outcomes of 189 patients with HCC less than 7 cm that had TACE plus RFA vs. RFA alone [34]. Zhang et al. then subsequently analyzed the 7-year outcomes of the entire cohort. TACE was performed with epirubicin and mitomycin emulsified into Lipiodol. An additional lipiodol injection was performed to slow antegrade flow if needed. RFA was performed within 2 weeks of TACE under US guidance. The combination group demonstrated significantly improved 5-year and 7-year OS and RFS. In the subgroup analysis, this held true for tumors larger than 3 cm [9].

The available literature on SIALI-guided thermal ablation, while limited in volume, demonstrates consistent signals across key outcome domains. Pooling the reported data, technical success rates across studies range from 93% to 100%. Local tumor recurrence or progression rates in SIALI cohorts were consistently lower than comparator arms where reported: Wu et al. demonstrated local tumor recurrence rates of 4.4%, 6.9%, and 7.5% at 1, 2, and 3 years in the SIALI group versus 14.4%, 16.3%, and 16.3% in the ultrasound-guided group (*p* = 0.002, 0.009, and 0.016, respectively). Kobe et al. reported 0% local recurrence at a mean follow-up of 3 years across all 20 lesions. Overall survival benefit was demonstrated in two studies (Adwan et al., Peng/Zhang et al.), though this did not reach significance in the remaining cohorts, likely reflecting differences in follow-up duration, tumor stage, and adjuvant therapies. These findings are summarized in Table 1 and must be interpreted in the context of the significant methodologic heterogeneity across studies, which precludes formal meta-analytic pooling [9,19,32,33,34,36].

## 6. Discussion

As described above, the SIALI technique and its institutional variations represent one component of the broader toolkit for curative-intent thermal ablation of hypervascular liver tumors. There are multiple mechanisms behind this, including embolization to decrease the heat sink, improve tumor demarcation for targeting, and optimize visualization for ablative margin assessment. Collectively, these properties suggest that Lipiodol may function as more than a conventional contrast agent in this context, potentially serving as a persistent imaging marker to support targeting and post-ablation assessment [19]. However, there is a diversity of treatment algorithms worldwide. Understanding that radioembolization and/or radiation segmentectomy is not a treatment option everywhere secondary to cost (single- or multi-vial) and/or regulatory approval, alternative techniques are needed to optimize outcomes.

Given the long survival rates of patients with very early and early-stage HCC, complete pathologic necrosis (CPN) has become a surrogate outcome measure in such patients. Additionally, the importance of CPN in long-term recurrence-free survival and overall survival post-transplant is well known [39,40]. In the United States, radiation segmentectomy with Y90 has emerged as a leading locoregional bridging strategy for HCC, driven in part by data demonstrating CPN rates approaching 100% with appropriate patient selection and dosimetry [41]. This shift also reflects a broader US practice evolution favoring transarterial over percutaneous approaches. This is influenced by proceduralist comfort, evolution of radioembolization dosimetry with access to dosimetry platforms, and an evolving recognition of the discordance between imaging complete response and true pathologic necrosis [41]. However, radiation segmentectomy and radioembolization in general are not universally available due to cost and regulatory constraints, and their routine use in this context is not the global standard. For many institutions worldwide, percutaneous ablation remains the primary curative-intent modality for appropriately selected patients and lesions. In this context, techniques such as SIALI and hepACAGA, that optimize targeting and ablation margin assessment, may help close the gap in achieving CPN rates with thermal ablation [41]. Crocetti et al. demonstrated CPN rates for MWA up to 78% [42]. However, CPN has been quoted anywhere from 57 to 95% [43]. It should be noted that CPN rates with thermal ablation are highly variable, likely reflecting differences in operator experience, patient selection, absence of real-time confirmation software, and access to post-ablation margin assessment tools [43]. Recently, Mosenthal et al. demonstrated a 71% CPN rate with TACE/ablation. The TACE technique was lipiodol emulsified with doxorubicin and mitomycin-C, and MWA was performed the same day or the next day. While the authors reported on a small number of patients, this information still informs us of the potential benefit of this combination/multimodal therapy strategy, especially in difficult-to-target lesions [44]. This is especially true considering MWA is also not ubiquitously available worldwide, and that RFA, while more available, has been associated with CPN rates from 26 to 96% [43].

The SIALI technique carries inherent procedural risks and technical limitations that warrant explicit discussion. As a catheter-based arterial procedure, it introduces risks associated with vascular access, including hematoma, arterial injury, and contrast nephropathy, in addition to the post-embolization syndrome (fever, pain, elevated liver enzymes) that may follow lipiodol injection, particularly when emulsified with chemotherapeutic agents. Lipiodol carries a known non-target embolization risk if selective catheterization is suboptimal, with potential for non-tumoral hepatic parenchymal ischemia. Hepatic arterial injury, including vasospasm or dissection during microcatheter manipulation, is a recognized though infrequent complication of selective hepatic arteriography. Specific to the technique, incomplete lipiodol retention, as demonstrated by Tan et al., is associated with inferior local recurrence-free survival, suggesting that the degree of tumor vascularity and lipiodol delivery are critical determinants of efficacy that cannot always be controlled by the operator [35]. Hypovascular tumors, including some metastatic histologies, may not retain lipiodol adequately for reliable targeting, as reflected by Smits et al.’s 7% failure rate attributable to this mechanism [18]. Anatomically, tumors in proximity to major hepatic veins or portal pedicles may have attenuated lipiodol retention due to washout, limiting the durability of tumor staining in delayed same-day or next-day ablation settings [35,45]. However, technique alterations can overcome tumoral hypovascularity, as shown by Miyayama et al.; the authors demonstrated marked lipiodol uptake in hypovascular HCCs with ultra-selective catheterization and injection [24]. Workflow complexity represents an additional practical limitation: the need for angiographic suite access, microcatheter expertise, and, in some cases, the lack of a hybrid angiography-CT environment introduces logistical barriers at institutions without integrated procedural suites or dedicated IR-oncology infrastructure. The existing literature does not systematically report complication data using standardized grading systems. Where adverse events were reported, they can be categorized by “likely etiology” as follows: (1) Arterial access-related: hematoma at the puncture site and arterial vasospasm or dissection during microcatheter manipulation, recognized though infrequent complications of selective hepatic arteriography. (2) Lipiodol/contrast-related: contrast nephropathy (relevant in patients with pre-existing renal dysfunction), non-target embolization if catheter positioning is insufficiently selective, and post-embolization syndrome (fever, pain, transient liver function test (LFT) elevation), which is more prominent when Lipiodol is emulsified with chemotherapeutic agents. Thyroid dysfunction is a recognized delayed complication of iodized oil administration and should be monitored per prescribing information guidance. (3) Ablation-related: pneumothorax (reported in both Huang et al. [33] and Takaki et al. [37]), hemorrhage, and portal vein thrombosis (Takaki et al. [37]). The single SIR grade 4 event reported by Kobe et al., a cardiac arrest, occurred during ablation of a paraganglioma metastasis and is attributable to catecholamine release from tumor manipulation, not to Lipiodol toxicity; this should not be interpreted as a SIALI-related complication [19]. (4) Procedural complexity: The requirement for angiographic suite access and microcatheter expertise introduces logistical barriers, and the combined angiographic and CBCT or CT-guided workflow carries greater radiation exposure than standard US-guided ablation alone. Radiation dose optimization using low-dose CBCT or CT protocols, pulsed fluoroscopy, and minimizing fluoroscopy time should follow ALARA principles. Formal dosimetry data were not systematically reported in the included studies, representing a gap in the current literature. Most series do not include formal safety endpoints as primary outcomes, and adverse event grading using standardized SIR or CIRSE classification was inconsistently applied across studies.

## 7. Patient Selection

Identifying appropriate candidates is critical to maximizing the benefit of SIALI-guided ablation. Patients most likely to benefit include those with hypervascular HCC or hypervascular metastases (e.g., neuroendocrine tumors) confirmed on cross-sectional imaging of hepatic arterially enhanced CBCT or CT, lesions that are occult or poorly conspicuous on non-contrast CT and standard B-mode ultrasound, tumor size within ablation critera range (generally ≤3–4 cm for single-session ablation, though combination approaches may extend this), preserved hepatic function (Child-Pugh A or selected B), adequate renal function to tolerate arterial contrast administration, absence of iodinated contrast hypersensitivity, and access to a hybrid angiography-CT or CBCT-capable angiography suite for simultaneous treatment strategies or CT suite access for sequential treatment strategies. As the technique may incidentally identify small daughter nodules or satellite lesions that were not conspicuous on preprocedural imaging, recognition may allow modification of the treatment strategy.

Patients less likely to benefit or in whom SIALI may be less appropriate include those with hypovascular tumors (e.g., colorectal liver metastases) where Lipiodol retention is unreliable, severely impaired renal function limiting contrast administration, known iodinated contrast hypersensitivity, tumors in locations precluding safe ablation regardless of visualization (e.g., immediately adjacent to critical structures without feasibility of hydrodissection), and settings where only conventional US guidance is available without angiographic capability. The evidence base is almost exclusively derived from HCC cohorts; generalizability to non-HCC histologies, including intrahepatic cholangiocarcinoma (which is often hypovascular on preprocedure imaging), is not fully supported by currently available data. However, work by Malavia et al. has shown that hypovascular tumors on pre-intervention imaging are often hypervascular on intraprocedure hepatic arterial enhanced CBCT or CT [46]. Additionally, ultraselective lipiodol delivery into hypovascular tumors can optimize lipiodol uptake, as shown by Miyama et al. [24]. As a result, future work is needed to determine efficacy in alternative and truly hypovascular tumor types, given the potential negative imprint, characterized by the relative absence of Lipiodol deposition within the tumor against an enhanced background of surrounding hepatic parenchyma. This negative contrast effect may provide visual guidance for thermal ablation, but its utility and outcomes are not known.

The hepACAGA technique has similarities to the SIALI technique in that a hepatic artery catheter is in place, allowing for multiple low-contrast-dose CBCTs as opposed to limited intravenous large-contrast-dose MDCTs. While tumor targeting may be performed with either technique via US, repeated CBCTs, or CBCT needle guidance, ablation confirmation requires CBCT fusion to the initial contrast-enhanced CBCT. Unfortunately, this is not available from all angiographic vendors, and third-party ablation confirmation software platforms currently do not support CBCT fusion. However, the value of the hepACAGA technique over traditional CT-guided thermal ablation cannot be understated. Smits et al. evaluated the combination of hepatic angiography and C-arm CT for CT-guided ablation (HepACAGA) in 21 patients with 28 tumors. Tumors were hepatocellular carcinoma, colorectal metastases, and neuroendocrine metastases. The technical success rate was 93%, as two lesions were too small and hypovascular to identify. They observed no local tumor recurrences at a median follow-up time of 16 months [18]. Wijnen et al. further summarized the added benefits of using C-arm integrated navigation software for needle trajectory planning, having the option to complete combination locoregional therapies or convert to a transarterial therapy entirely for the same or additional lesions (e.g., TACE, Y90), blocking heatsink via arterial occlusion, and allowing for immediate endovascular treatment of complications of ablation such as arterial injury and hemorrhage. This technique provides great flexibility in treatment options for single or multi-session therapy [18,47]. Additionally, compared to traditional US/CT-guided thermal ablation, Wijnen et al. evaluated 121 patients between the 2 cohorts retrospectively. Their work encompassed both HCC and CRLM but showed that both local recurrence and local progression-free survival were statistically significantly improved in the hepACAGA group. Additionally, complication rates were significantly improved in favor of this technique [48]. Resultantly, the hepACAGA technique represents a significant step forward in the thermal ablation treatment algorithm compared to conventional techniques. However, the SIALI technique may be more ubiquitously available for utilization.

Odisio et al. recently published the COVER-ALL trial. In a prospective phase 2 randomized study, 50 patients were evaluated in two groups: the experimental group had software-assisted evaluation of the ablative margin, while the control group had visual assessment of the ablative margin. This resulted in a mean ablative margin of 5.9 mm in the experimental group and 2.2 mm in the control group, achieving statistical significance. As a result, the control was halted while the experimental group continued. The impact of software assistance, or ablation confirmation platforms, has, as a result, been solidified with historic and traditional visual assessment falling out of favor. Unfortunately, as with other technologies, cost and regulatory approval again limit the implementation of such software platforms [15]. As shown by Hu et al., the ablative margin can be manually measured using the Lipiodol-stained tumor as a reference when using the SIALI technique [49]. It is important to note that direct comparative evidence between SIALI-guided visual margin assessment and validated ablation confirmation software platforms does not currently exist, and SIALI should not be positioned as an equivalent alternative. Rather, this technique may serve as a clinically useful adjunct in settings without access to such platforms, while these technologies become more widely available. Resultantly, this technique may allow time for clinical practices to bridge to the implementation of ablation confirmation software when more widely available, whether for CT or CBCT.

The existing body of evidence supporting SIALI-guided thermal ablation is subject to several important methodologic limitations that constrain the strength of conclusions that can be drawn. The majority of published studies are retrospective single or multi-center cohort analyses, which are susceptible to selection bias in that patients selected for the combination technique may represent favorable tumor or patient profiles not fully captured by propensity score matching. Cohort sizes are modest, with most series enrolling fewer than 200 patients, and the single prospective randomized controlled trial (Peng et al.) incorporated TACE rather than bland lipiodol injection, limiting direct applicability to the SIALI technique as described herein [34]. Outcome heterogeneity across studies reflects inconsistent definitions of technical success, local recurrence, and ablation margin adequacy, further limiting cross-study comparisons. Follow-up durations vary substantially across cohorts, and long-term oncologic outcomes beyond 3–5 years are not available for most series. The studies reviewed are predominantly conducted in Asian patient populations with HCC arising in the context of viral hepatitis, and generalizability to Western populations with metabolic-associated liver disease or to non-HCC histologies is uncertain. Critically, no prospective comparative studies directly comparing SIALI to standard CT-guided or US-guided ablation without arterial augmentation exist, with local recurrence as a pre-specified primary endpoint and powered accordingly. The absence of standardized lipiodol preparation protocols across studies (lipiodol alone versus emulsified formulations with varying chemotherapeutic agents) introduces an additional confounding variable that makes attribution of outcomes to lipiodol-guided targeting versus pharmacologic or embolic effects difficult to isolate.

## 8. Conclusions

SIALI-guided thermal ablation is a technically feasible adjunct technique with a plausible mechanistic rationale and early supportive clinical signals for its use in hypervascular liver tumors, particularly in cases of limited lesion conspicuity on standard imaging and at institutions where ablation confirmation software is unavailable. The published literature, comprising predominantly retrospective cohorts with modest sample sizes, heterogeneous technical approaches, and variable follow-up, suggests favorable technical success and safety as well as signals of improved local tumor control in selected patient populations. However, the current evidence base is insufficient to establish SIALI as a standard of care, and conclusions regarding long-term oncologic benefit remain preliminary given the methodologic limitations described. Prospective comparative studies with standardized Lipiodol protocols, pre-specified ablation margin endpoints, and adequate follow-up are needed to define the role of this technique within the broader locoregional therapy algorithm. In the interim, SIALI may serve as a practical and accessible option for centers seeking to optimize ablation targeting and margin assessment, with integration of AI-assisted planning and ablation confirmation platforms representing an area warranting future investigation.

## Figures and Tables

**Table 1:** Study Design and Technical Details for Published Studies on SIALI-Guided Thermal Ablation for Hepatic Malignancies [9,19,32,33,34,35,36,37].

Study (Author, Year)	Design	N Patients/N Lesions	Tumor Type	Tumor Size (Mean/Median, Range)	Lesion Visibility Pre-Lipiodol	Comparator Group	Lipiodol Prep/Volume	Chemo-Therapy	Interval (Lipiodol → Ablation)
Wu et al., 2021 [36]	Retro cohort (PSM)	320 pts (160 IoCT vs. 160 US)	HCC (within Milan criteria)	Mean 2.2 ± 0.9 cm; >80% ≤3 cm	NR; Lipiodol used for guidance	US-guided RFA	Lip alone; 2–5 mL (size-dependent)	None	SS (angio then CT same day)
Kobe et al., 2023 [19]	Retro cohort	18 pts/20 lesions	HCC (25%); liver mets (75%: NET, adrenal, thyroid, paraganglioma, GIST)	Median 1.5 cm (1–2.5 cm)	All invisible on US & NCCT; visible only on arterial-phase MRI/CT	None (single arm)	Lip:saline emulsion (3:1); median 3 mL (1–10 mL)	None	SS (hybrid angio-MDCT suite)
Tan et al., 2022 [35]	Retro cohort (lesion-level PSM)	198 pts/280 lesions (121 pairs post-PSM)	HCC	NR as single mean; subgroups ≤ 3 cm vs. >3 cm, majority ≤ 3 cm	NR	Complete vs. incomplete Lipiodol retention (internal)	Lip alone OR lip-epirubicin/doxorubicin emulsion; up to 15 mL lip + 50–120 mg epirubicin or 100 mg doxo	Variable: TACE (n = 218) or TLI alone (n = 62)	Variable: within 1 mo (n = 192) or >1 mo (n = 88)
Huang et al., 2020 [33]	Retro comparative	118 pts (59/grp); 270 tumors (144 TAE+RFA, 126 RFA-alone)	HCC (recurrent/residual)	10–30 mm; 71 tumors ≤ 2 cm; 47 > 2–3 cm	Retained iodized oil from prior TACE used as a landmark; direct visibility NR	CT-guided RFA	Lip alone via hepatic artery	None (bland TAE only)	Sep sessions; median 0.8 d (0–2 d)
Adwan et al., 2023 [32]	Retro comparative	112 pts (49 TAE + MWA, 63 MWA alone); 122 lesions (55/67)	HCC (within Milan criteria)	Mean 1.9 ± 0.7 cm (TAE + MWA); 2.0 ± 0.8 cm (MWA alone)	NR	MWA	Lip alone; up to 10 mL (to blood stasis)	None (bland TAE only)	Sep session; MWA ≥ 24 h after TAE
Peng et al., 2013/Zhang et al., 2021 [9,34]	RCT + long-term follow-up	189 pts (94 TACE + RFA, 95 RFA alone)	HCC < 7 cm	Mean 3.47 ± 1.44 cm (TACE + RFA); 3.39 ± 1.35 cm (RFA alone)	All visible on US (required for inclusion)	RFA alone	Lip-epirubicin/mitomycin emulsion (epirubicin 50 mg + mitomycin 8 mg in 5 mL lip)	Yes: epirubicin + mitomycin (conventional TACE)	Sep sessions; RFA within 2 wk of TACE (median 7 d, 3–14 d)
Takaki et al., 2013 [37]	Retro cohort	67 pts/150 lesions	HCC	Mean 1.3 ± 0.6 cm (0.5–4.2 cm)	All invisible on US (isoechoic/hepatic dome); all visible on CT fluoro post-lipiodol	None (single arm)	Lip alone; 1–10 mL (mean 4.0 ± 1.8 mL); cisplatin 100 mg added in 7 palliative pts	None (curative arm); cisplatin in some palliative pts	Sep sessions; within 1 wk (mean 1.5 ± 2.6 d)

**Table 2:** Outcomes for Published Studies on SIALI-Guided Thermal Ablation for Hepatic Malignancies [9,19,32,33,34,35,36,37].

Study (Author, Year)	Ablation Modality	Intended Ablative Margin	Follow-Up (Mean/Median)	Primary Endpoint	Key Outcome(s)
Wu et al., 2021 [36]	RFA	NR	Mean ~49 mo	LTP rate	1/2/3-yr LTP: 4.4/6.9/7.5% (IoCT) vs. 14.4/16.3/16.3% (US), *p* = 0.002; improved RFS, no OS difference
Kobe et al., 2023 [19]	RFA (cluster/expandable), MWA, CRA	5 mm	Mean 3 ± 2.5 yr	Technical success (visualization + ablation)	100% technical success; 0% local recurrence at mean 3-yr follow-up
Tan et al., 2022 [35]	MWA (82.5%) or RFA (17.5%); all CT-guided	5 mm	NR; OS median not reached	LRFS	Complete retention: median LRFS 1279 d vs. 819 d (incomplete), *p* = 0.030; no OS difference (*p* = 0.456)
Huang et al., 2020 [33]	CT-guided RFA (Cool-tip, 2- or 3-cm active tip)	5 mm	Mean 13.2 mo (3–36.5 mo)	LTP rate	ORR 96.61% vs. 79.66% (*p* = 0.008); 1-yr LTP lower in TAE + RFA group; median time to LTP 9.6 vs. 4.8 mo
Adwan et al., 2023 [32]	CT-guided MWA	5 mm	OS curves extend to ~140 mo; median NR	OS	12/24/36-mo OS: 97.7/85.1/78.8% (TAE + MWA) vs. 91.9/71.4/59.8% (MWA), *p* = 0.004; LTP: 5.5% vs. 7.5%, *p* = 0.73 (NS)
Peng et al., 2013/Zhang et al., 2021 [9,34]	US-guided RFA (LeVeen expandable electrode)	NR	Median 56 mo (TACE + RFA), 50 mo (RFA); censored Dec 2019	OS; RFS (long-term follow-up)	5/7-yr OS: 52.0/36.4% (TACE + RFA) vs. 43.2/19.4% (RFA), HR 0.55, *p* = 0.001; benefit primarily in tumors > 3 cm
Takaki et al., 2013 [37]	CT fluoroscopy-guided RFA (Cool-tip internally cooled electrode)	5 mm (exceptions for lesions adjacent to major vessels or diaphragm)	Mean 23.2 ± 18.0 mo (1.8–74.7 mo)	Technical success, LTP, survival	100% technical success; 1/3/5-yr LTP: 3.9/5.3/5.3%; median OS 33.4 mo (curative 34.7 vs. palliative 11.8 mo)

Abbreviations (Table 1 and Table 2): CRA = cryoablation; CT = computed tomography; GIST = gastrointestinal stromal tumor; HCC = hepatocellular carcinoma; IoCT = iodized oil CT-guided; Lip = Lipiodol; LTP = local tumor progression; LRFS = local recurrence-free survival; MWA = microwave ablation; NCCT = non-contrast CT; NET = neuroendocrine tumor; NR = not reported/not explicitly stated; NS = not significant; OS = overall survival; PSM = propensity score matching; RCT = randomized controlled trial; Retro = retrospective; RFA = radiofrequency ablation; RFS = recurrence-free survival; Sep = separate; SIALI = selective intra-arterial lipiodol injection; SS = same session; TACE = transarterial chemoembolization; TAE = transarterial embolization; TLI = transarterial lipiodol injection; US = ultrasound.

## Data Availability

For this type of study, the availability of data and materials is publicly available, as this is a narrative review.
